# DEPRESSION AMONG OLDER ADULTS WITH ACUTE CORONARY SYNDROME ASKING FOR EMERGENCY HELP

**DOI:** 10.1093/geroni/igad104.2434

**Published:** 2023-12-21

**Authors:** Audai Hayajneh

**Affiliations:** Jordan University of Science and Technology, Irbid, Irbid, Jordan

## Abstract

Acute coronary syndrome (ACS) has been associated with urgent cardiac symptoms, which require an emergent medical care by the healthcare providers (HCPs), including nurses. These cardiac emergency events may cause disturbance to daily life for older adults with ACS. The purpose of this study was to identify the occurrence of depression and examine its correlates among those patients with ACS asking for emergency help. This is secondary data analysis of data collected from a convenient sample of 300 older adults with ACS asking for emergency help. Correlation and multivariate analyses were utilized in this study. Depression was found to be significantly, positively associated with each of hospital length of stay (LOS), intensive care unit (ICU) LOS, and frailty. The level of depression among older adults with ACS asking for emergency help was 65.7%. The level of depression among older adults asking for emergency help had significant predictors, as follows: Age (t=3.06), troponin (t=2.98), hemoglobin alpha 1C (HbA1c) (t=3.18), and frailty (t=5.77). These factors utilized in the linear regression model explained 56.6% (Adjusted R²=0.566, p=0.017) of the variation in the level of depression. The rate of depression is high among older adults asking for emergency help. Thus, the HCPs, including nurses, should pay a high attention to those at-risk patients for the occurrence of depression, as well as be well-prepared to manage correspondingly. Depression among older adults with ACS asking for emergency help requires emergency intervention protocol to combat depression among those patients by HCPs, including nurses.

